# Association between social integration and loneliness among the female migrant older adults with children: the mediating effect of social support

**DOI:** 10.1186/s12877-023-04569-8

**Published:** 2024-01-03

**Authors:** Jing Xu, Guangwen Liu, Hexian Li, Xiaoxu Jiang, Shengyu Zhou, Jieru Wang, Mingli Pang, Shixue Li, Fanlei Kong

**Affiliations:** 1 Centre for Health Management and Policy Research, School of Public Health, Cheeloo College of Medicine, Shandong University, Jinan, 250012 China; 2 NHC Key Lab of Health Economics and Policy Research, Shandong University, Jinan, 250012 China; 3 Department of Medical Administration, The First Affiliated Hospital of Anhui Medical University, Hefei, 230000 China; 4 Institute of Health and Elderly Care, Shandong University, Jinan, China

**Keywords:** Migrant older adults with children, Female, Loneliness, Social integration, Social support, Structural equation model

## Abstract

**Background:**

The number of migrant older adults with children (MOAC) in China has been increasing in recent years, and most of them are women. This study aimed to explore the mediating effect of social support between social integration and loneliness among the female MOAC in Jinan, China.

**Methods:**

In this study, 418 female MOAC were selected using multi-stage cluster random sampling in Jinan, Shandong Province, China. Loneliness was measured by the eight-item version of the University of California Los Angeles Loneliness Scale (ULS-8), and social support was measured by The Social Support Rating Scale (SSRS). Descriptive analyses, *t*-tests, ANOVA, and structural equation modeling (SEM) were used to illustrate the relationship between social integration, social support, and loneliness.

**Results:**

The average scores of ULS-8 and SSRS were 12.9 ± 4.0 and 39.4 ± 5.9 among female MOAC in this study. Social integration and social support were found to be negatively related to loneliness, and the standardized direct effect was -0.20 [95% CI: -0.343 to -0.068] and -0.39 [95% CI: -0.230 to -0.033], respectively. Social support mediated the relationship between social integration and loneliness, and the indirect effect was -0.16 [95% CI: -0.252 to -0.100].

**Conclusion:**

The female MOAC’s loneliness was at a relatively lower level in this study. It was found that social integration was negatively associated with loneliness, and social support mediated the relationship between them. Helping female MOAC integrate into the inflow city and improving their social support could be beneficial for alleviating their loneliness.

## Background

As the world’s most populous country, China has a very large group of elderly population. According to data from the Seventh National Census, in 2020, the total number of people aged 65 and above in China was about 191 million, accounting for about 13.5% of the total population [1]. Because of urbanization and industrialization, large-scale population migration movements have emerged in China since the last century [2]. In China, there were 376 million internal migrants whose homes were not the locations of household registration (hukou) in 2020, with 249 million of them moving from rural to urban areas and 127 million from urban to urban areas, respectively [3]. Driven by the rapid development of aging, the number of migrant elderly people in China increased year by year [4]. The 2018 report on China’s migrant population development showed that the number of Chinese migrant elderly increased from 5.03 million in 2000 to 13.04 million in 2015 [5]. Most Chinese elderly migrants move to cities where their children live to provide care for their grandchildren [6]. In the current study, these elderly are referred to as the migrant older adults with children (MOAC) [7]. In general social and cultural norms, women are primarily responsible for childcare and other family responsibilities [8], while most grandchild care is also provided by grandmothers in current Chinese family [9, 10], which result in more women than men among the migrant elderly in China [11].

Loneliness has become a common problem among older people worldwide [12]. J de Jong-Gierveld’s understanding of loneliness emphasized more on subjective feelings, and considered loneliness as an unpleasant emotional experience resulting from the perception of social isolation or lack of contact with others [13]. Peplau et al. consider loneliness as subjective social isolation, an unpleasant experience that arises when there is a discrepancy between an individual’s desired social relationships and the reality [14]. A study in Anhui, China showed that 78.1% of older people had moderate to severe levels of loneliness [15]. In a study among Finland’s elderly, about 39% of the older people felt lonely [16]. Factors associated with loneliness in older adults, such as sociodemographic characteristics [17, 18], social participation [19], social support [20], smoking [21], and physical exercise [22] were identified in some studies. Concerning the female elderly, about half of the Indonesian older women always felt lonely [23]. Widowhood, depression, mobility problems, and mobility reduction increased the risk of loneliness among older women in Sweden [24]. Several studies also showed that the female elderly reported higher levels of loneliness than males [25, 26]. Although there are more studies on loneliness among the older people, little attention has been paid to the loneliness of female migrant older adults in China.

Social integration is defined as participation in a wide range of social relationships [27], which is an essential element of health [28] and is considered to be related to loneliness for older people [29]. A study in Korea showed that the migrant elderly with better social integration had lower levels of loneliness [30]. A study on internal migrant workers in China found that the social integration of migrant workers was significantly and negatively related to loneliness [31]. Santini’s study on older Americans showed that when older adults were more socially disconnected, they perceived higher levels of loneliness [32]. Jang et. al’s study on Korean American older immigrants found that social integration was negatively related to loneliness [30]. Many studies have examined the social integration of migrant populations, but there is still a lack of attention to the social integration of female migrant older adults, with some studies in China mainly focusing on their health status [33–35].

Social support comes from people’s social needs, and typically refers to services, care, or encouragement provided by members of social networks [36]. Previous studies have shown that social support was negatively related to loneliness [37–39]. Among Chinese older adults, social support from family or friends could help alleviate their loneliness [39]. Wong and Leung’s study showed that social support had a positive impact on the mental health of Chinese female migrants [40], however, the study did not further explore the relationship between social support and other factors, such as social integration. A study of Muslim elderly in Turkey suggested that perceived social support was an important factor influencing loneliness in older adults [38].

Social integration and social support are both important and unique aspects of social relationships. Social integration referred to the principle of how individuals related to each other at the society level, and also reflected the connection between the individual and society, the community or other units [41]. Social support, on the other hand, emphasizes the real or perceived help and support that individuals receive from their social networks in difficult times [42]. Social integration mainly focused on interpersonal interactions within the background of social structures, and transferring individual characteristics and behaviors to the environment or group. For example, the community, which is an important environment for people’s social integration. While social support was mostly still a concept at the individual level, focusing on interpersonal interactions only [43, 44]. There was little research on the relationship between social support and social integration, although social support and social integration could jointly influence an individual’s health [45, 46]. A study showed that when the old adults’ social networks were limited, the level of social support generally decreased [47].

In summary, previous studies had explored the effect of social integration on loneliness, as well as the effect of social support on loneliness, yet none study clarified the relationship between social integration, social support, and loneliness simultaneously, not mention among the female MOAC. Thus, this study aimed to clarify the association between social integration and loneliness and the mediating role of social support between them among the female MOAC in Jinan, China.

## Methods

### Study design and participants

This study is a cross-sectional survey conducted in Jinan City, Shandong Province, China, in August 2020. Jinan is the capital city of Shandong Province, with 10 districts and 2 counties under its jurisdiction. In this study, multi-stage cluster random sampling was used to extract the survey sample, taking into account the geographical location and economic development of each district in Jinan. First, we selected three districts from the 10 districts in Jinan as primary sampling units (PSUs), then we selected one street from each of the three primary sampling units as a secondary sampling unit (SSUs); finally, we selected one community from each of the three secondary sampling units, so three communities in Jinan City were finally selected as the research sites. The survey was conducted among the elderly in these three communities who were 60 years old or older and had migrated to live in Jinan following their children, and those who were female in the survey became the target group of this study.

The investigators were thirty-two college students who were trained on the survey background, questionnaire content, and conversation skills before conducting the door-to-door interview. Finally, a total of 656 valid migrant elderly samples were obtained, of which 418 female migrant elderly were selected in this study.

### Measurements

#### Dependent variable: loneliness

Loneliness was measured using the eight-item version of the University of California Los Angeles Loneliness Scale (ULS-8), this scale is a unidimensional scale with 8 items, which consists of 6 “lonely” positive ordinal items and 2 “non-lonely” inverse ordinal items, each of the items is scored on a 4-point Likert scale [48]. The total score on the scale ranged from 8 to 32, and the higher the total score, the higher the degree of loneliness. The scale has been widely used and has good reliability among Chinese older adults [49]. The Cronbach’s α for this scale in this study was 0.817.

#### Independent variables

##### Social support

Social support of female MOAC was measured by the Social Support Rating Scale (SSRS), The scale was developed by Xiao [50] in 1986 and consisted of ten items with three dimensions: objective support, subjective support, and availability. The SSRS has been widely used among Chinese people and has good reliability and validity [51]. The Cronbach’s α for SSRS in this study was 0.723.

##### Social integration

Following previous studies [52–55], social integration was assessed by four domain questions: economic integration, psychological integration, cultural integration, and community integration. Each domain contained one question: the question “How much is your monthly income?” is for economic integration; “Do you think you have become a local?” for psychological integration; “Can you speak the local Chinese dialect?” for cultural integration, and “How often do you participate in community activities?” for community integration.

##### Covariates

Sociodemographic characteristics mainly included age, education level, and marital status; other variables included chronic disease, and self-rated health.

### Analysis approach

Descriptive statistics were used to describe the sociodemographic characteristics, health status, and social integration of the female MOAC. T-test and ANOVA were employed to compare the statistical differences in loneliness among the female MOAC with different characteristics. A *p*-value of < 0.05 denoted statistical significance, and all the analyses were performed using SSPS 24.0.

Structural equation modeling (SEM) was used to test whether social support mediates the relationship between social inclusion and loneliness. A 95% confidence interval (95% CI) of the estimated standardized effects was determined using the bootstrap method with 2,000 samples [56]. The indirect effect was regarded as statistically significant if the 95% CI excluded zero [56]. The fit of the structural equation model took into account several indicators. Good model fit was accepted when χ^2^/df < 3.00, root mean square error of approximation (RMSEA) ≤ 0.08, incremental fit index (IFI) ≥ 0.900, goodness-of-fit index (GFI) ≥ 0.900, adjusted goodness-of-fit index (AGFI) ≥ 0.900, and comparative fit index (CFI) ≥ 0.900 [57–59]. All SEM analyses were performed using AMOS 24.0.

## Results

### Basic characteristics of the participants

Table 1 showed the general demographic characteristics of female MOAC. Nearly half (48.1%) of the 418 female MOAC were between the ages of 60 and 65, and more than 60% of them had an elementary school education level or less. 88% of the female MOAC were unmarried, and more than 40% had at least one chronic disease. 57.2% of the participants had a monthly income of less than 500 RMB, and only 23.2% had an income of 1000 RMB or more. Almost half of female MOAC (44.7%) could just understand but couldn’t speak the local dialect, while 52.2% of them never participated in community activities.

**Table 1:** The basic characteristics of the female MOAC in this study

Characteristics	Categories	N	%
**Total**		418	100
**Age**	60–65	201	48.1
	66–70	207	51.9
**Education**	Illiterate	158	37.8
	Elementary school	103	24.6
	Middle School	102	24.4
	High School and above	55	13.2
**Marital status**	Married	368	88.0
	Unmarried*	50	12.0
**Chronic diseases**	No	243	58.1
	Yes	175	41.9
**Self-rated health**	Excellent	104	24.9
	Very good	144	34.4
	Good	81	19.4
	Fair and below	89	21.3
**Monthly income**	0–100 RMB	157	37.6
	101–500 RMB	82	19.6
	501–1000 RMB	82	19.6
	1001–2500 RMB	53	12.7
	2501–10000 RMB	44	10.5
**Feel like a local**	Don’t agree	153	36.6
	Fair	172	41.1
	Agree	93	22.2
**Local Chinese dialect**	Can’t understand or speak	24	5.7
	Can understand but can’t speak	187	44.7
	Can speak a little	114	27.3
	Can fluently speak	93	22.2
**Participate in community activities**	Never	218	52.2
	Occasionally	87	20.8
	Sometimes	55	13.2
	Often	58	13.9

^*^:“Unmarried” includes: divorced, single, and widowed

### Social support and loneliness of the female MOAC

Table 2 demonstrated the social support and loneliness of the female MOAC in this study. As for social support, the mean score of SSRS among total female MOAC was 39.4 ± 5.9. Among all age groups, those female MOAC aged 60–65 years old had the highest mean score of social support (40.0 ± 6.0). The mean score of social support was statistically significantly higher among those with a spouse (39.9 ± 5.7) than those without a spouse (35.2 ± 5.9, *p* < 0.001). The female MOAC had a high level of social support (41.1 ± 6.6) when they felt they had become local people. When they participated in community activities frequently, they had a higher social support score compared to those who never participated in community activities (41.9 ± 6.7 V.S. 38.2 ± 5.6). There was a significant difference in the social support scores of female MOAC with a different frequency of participation in community activities (*p* < 0.001).

**Table 2:** Social support and loneliness of the female MOAC in this study

Characteristics	Social Support		Loneliness	
	Mean (SD)	*p*	Mean (SD)	*p*
**Total**	39.4(5.9)		12.9(4.0)	
**Age**		0.001^a^		0.491^a^
60–65	40.0(6.0)		13.2(3.9)	
Over 65	38.9(5.6)		12.7(4.2)	
**Education**		0.096^a^		0.233^a^
Illiterate	39.2(6.0)		13.4(4.2)	
Elementary school	39.0(5.2)		12.8(3.9)	
Middle School	39.0(6.2)		12.5(4.0)	
High School and above	41.2(6.0)		12.4(3.5)	
**Marital status**		< .001^b^		0.404^b^
Married	39.9(5.7)		12.9(4.0)	
Unmarried	35.2(5.9)		13.4(4.0)	
**Chronic diseases**		0.020^b^		0.280^b^
No	39.9(5.7)		12.7(3.8)	
Yes	38.6(6.0)		13.2(4.2)	
**Self-rated health**		0.172^a^		0.595^a^
Excellent	40.5(5.1)		12.7(4.2)	
Very good	38.7(6.8)		13.3(3.9)	
Good	39.4(5.2)		12.7(4.1)	
Fair and below	39.0(5.6)		12.9(3.9)	
**Monthly income**		< 0.001^a^		< 0.001^a^
0–100 RMB	38.6(6.1)		13.2(4.2)	
101–500 RMB	38.2(6.2)		14.4(4.3)	
501–1000 RMB	39.4(4.8)		12.5(3.4)	
1001–2500 RMB	40.3(4.6)		11.3(3.1)	
2501–10000 RMB	43.2(6.1)		12.2(3.8)	
**Feel like a local**		< 0.001^a^		< 0.001^a^
Don’t agree	37.9(5.7)		13.7(4.5)	
Fair	39.7(5.3)		12.3(3.7)	
Agree	41.1(6.6)		10.9(2.9)	
**Local Chinese dialect**		0.010^a^		0.073^a^
Can’t understand or speak	36.9(7.3)		13.7(5.2)	
Can understand but can’t speak	38.9(5.0)		13.4(4.2)	
Can speak a little	40.7(5.7)		12.4(3.8)	
Can fluently speak	39.4(7.0)		12.4(3.3)	
**Participate in community activities**		< 0.001^a^		0.001^a^
Never	38.2(5.6)		13.0(4.0)	
Occasionally	40.1(5.5)		14.0(4.6)	
Sometimes	40.1(5.6)		12.6(3.4)	
Often	41.9(6.7)		11.3(2.7)	

^a^ANOVA

^b^t test

For loneliness, the average score of ULS-8 among female MOAC was 12.9 ± 4.0. Concerning the age difference, the highest mean score of loneliness was found among those aged 60–65 years old (13.2 ± 3.9). As for the incomes, those who had a monthly income of 101–500 RMB felt the highest level of loneliness (14.4 ± 4.3), and there was a statistically significant difference in loneliness among the elderly with different income levels (*p* < 0.001). The mean score of loneliness for female MOAC who agreed that they had become local residents (10.9 ± 2.9) was 2.8 points lower than those who disagreed (13.7 ± 4.5).

### The structural equation modeling analysis

#### Model fit indices

**Fig. 1:**
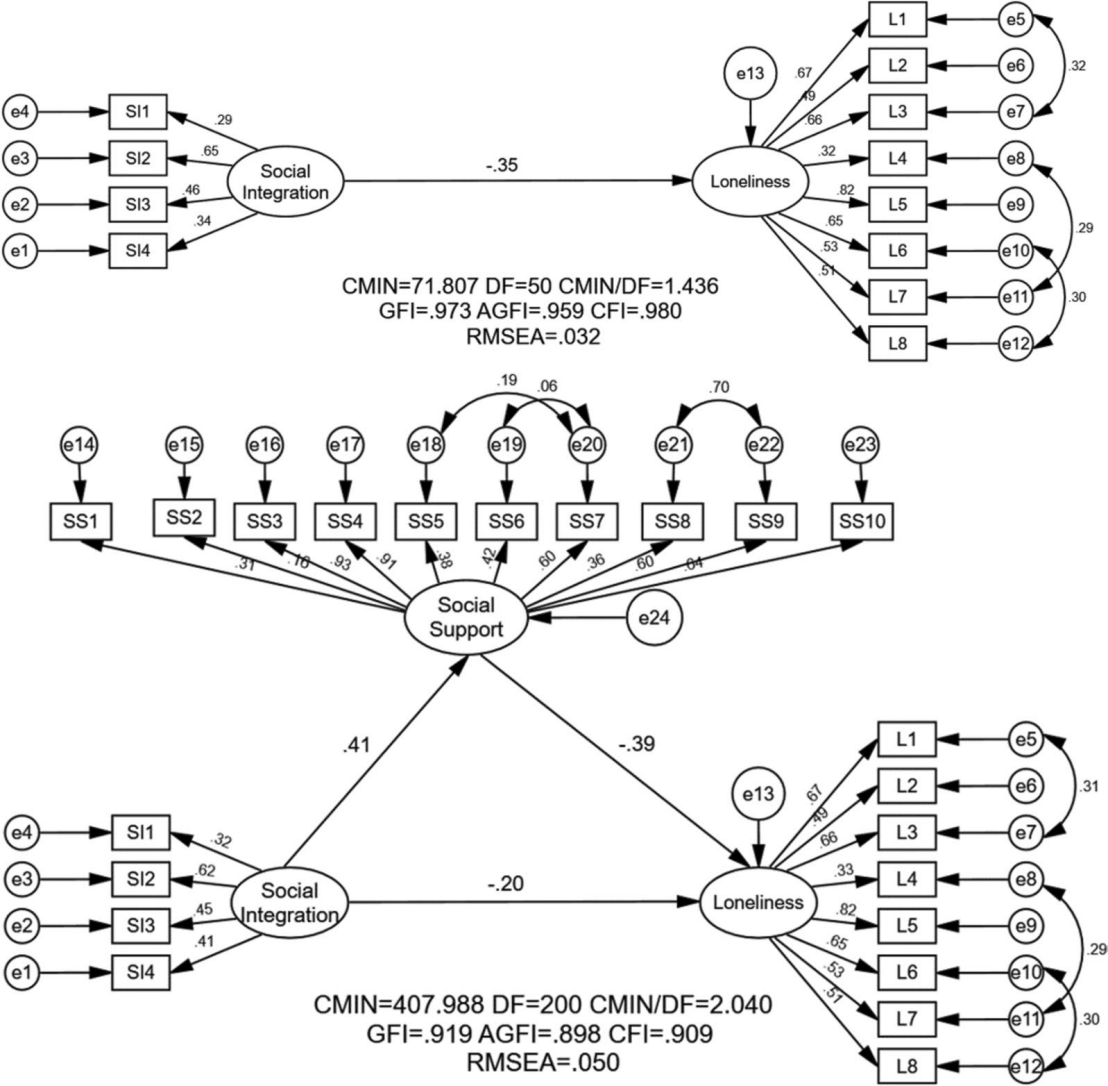
SEM analysis of the association between social integration and loneliness with social support as a mediator among the female MOAC in Jinan, China. Note: CMIN, χ^2^ value; AGFI, adjusted good-ness fit index; DF, degree of freedom; GFI, goodness fit index; AGFI, adjusted goodness fit index; CFI, comparative fit index; RMSEA, root mean square error of approximation. L1- L8, the items of ULS-8; SS1-SS10, the items of SSRS; SI1, economic integration; SI2, psychological integration; SI3, cultural integration; SI4, community integration

 Before using SEM analysis to explore whether social integration had a mediating effect between loneliness and social support, the effects of social support and social integration on loneliness were explored using multiple linear regression. The results indicated that all four dimensions of social integration and social support were influencing factors of loneliness, controlling for covariates (Appendix, Table 5). Figure 1 displayed the findings of SEM analysis of the study’s recommended two default models. The upper one in Fig. 1 showed a model of the total effect of social integration on loneliness (Model 1), while the lower one in Fig. 1 illustrated a mediated model that included social support (Model 2). It was found that social integration and social support were both related to loneliness, and social support mediates the relationship between social integration and loneliness.

Table 3 demonstrated the indicators related to the fit of Model 1 and Model 2, and it could be seen that the two models both fitted well. For the total effects model (Model 1), the χ^2^/df = 1.436, GFI = 0.973, CFI = 0.980, AGFI = 0.959, and RMSEA = 0.032, while for the mediated effects model (Model 2), χ^2^/df = 2.040, GFI = 0.919, AGFI = 0.898, CFI = 0.909 and RMSEA = 0.050.

**Table 3:** The model fit indicators

Model	CMIN	DF	CMIN/DF	GFI	AGFI	CFI	RMSEA	Decision
Cut-off criteria	**-**	**-**	** < 5**	** ≥ 0.900**	** ≥ 0.900**	** ≥ 0.900**	** ≤ 0.080**	
Model 1	71.807	50	1.436	0.973	0.959	0.980	0.032	Good fitting
Model 2	407.988	200	2.040	0.919	0.898	0.909	0.050	Good fitting

#### The mediating effect of social support on the association between social integration and loneliness

Table 4 revealed the mediating role that social support played between social integration and loneliness, and the upper and lower bounds of the bootstrap indicated that the mediating effect (indirect effect in Table 4) was statistically significant. When social support was not included in the model, the standardized total effect coefficient of social integration on loneliness was -0.36 (bootstrap 95% CI = -0.494 to -0.234). When social support was included as a mediating variable, the direct effect of social integration on loneliness was -0.20 (bootstrap 95% CI = -0.343 to -0.068), and the standardized indirect effect coefficient was -0.16 (bootstrap 95% CI = -0.252 to -0.100), with a mediating effect of 44.4%. Therefore, social support was determined to have a partially mediating role in the association between social integration and loneliness.

**Table 4:** The mediating effect of social support on the association between social integration and loneliness

Effect Type	Coefficient	BOOT CI lower	BOOT CI upper	Percentage (%)
**Direct effect**				55.6
Social integration → loneliness	-0.20	-0.343	-0.068	
Social support → loneliness	-0.39	-0.510	-0.271	
Social integration → Social support	0.41	0.267	0.567	
**Indirect effect**				44.4
Social integration → Social support → Loneliness	-0.16	-0.252	-0.100	
**Total effect**	-0.36	-0.494	-0.234	100

## Discussion

Female MOAC are a vulnerable group that had received little attention and loneliness is a common problem among them which is often influenced by social integration and social support. Our study was the first to use SEM to explore the relationship between social integration, social support, and loneliness among female MOAC, which could provide suggestions for future interventions for the female MOAC. This study found that social integration had a positive effect on social support and was negatively associated with loneliness; besides, social support mediated the relationship between social integration and loneliness among the female MOAC in Jinan, China.

### Loneliness of the female MOAC

The mean score of the ULS-8 of female MOAC was 12.9 ± 4.0 in this study, which was less than a study that found that the mean ULS-8 score was 15.5 ± 4.4 for female Chinese empty nesters in Liaoning Province, China [60], indicating the loneliness of female MOAC in this study was lower than the female empty nesters in China. The possible reason may be because unlike the female empty-nest elderly, the female MOAC were generally living together with their children and got more accompanying which could lower their feeling of loneliness [61]. Besides, the mean score of the ULS-8 of female MOAC in this study (12.9 ± 4.0) was also found to be higher than a previous study that included both the Chinese male and female MOAC (loneliness score was 12.82 ± 4.1) [62]. The possible reason may come from the fact that women were generally more emotional and sensitive than men.

### Association between social integration and loneliness

It was found that social integration was negatively correlated with the loneliness of female MOAC, which was consistent with David’s research on social integration and loneliness in later life [63]. A qualitative study on migrant elders in Jiangsu Province, China found that poorly integrated older people were more likely to feel lonely and desperate in a new city [64]. Domènech et al.’s study indicated that good social integration could alleviate the social isolation of the elderly and improve their mental health [65]. However, a qualitative research on the MOAC showed that they had a low level of social integration due to limited social interaction, increased intergenerational conflict, language barriers, and discrimination [66]. Thus, it is important to help the female MOAC better integrate into the new living environment to protect their mental health.

### Relationship between social support and loneliness

The results of the present study showed that higher levels of social support were associated with lower loneliness in female MOAC [67], which was consistent with Hom et al.’s study [68]. Another study of elderly widowed women in the United States of America showed that those with better social support had lower levels of loneliness [69]. For Chinese older adults, social support was found could relieve their loneliness; the most important social support was support from family members, and those elderly with poor family functioning would experience higher levels of loneliness [39]. Moreover, the negative association between loneliness and social support was stronger among the rural Chinese populations than the urban ones [70]. Since most of the female MOAC were from rural areas [71], more attention was needed on the social support from the family members to relieve their loneliness.

### Mediating effect of social support

The results of SEM analysis showed that social support could mediate the relationship between social integration and loneliness. When the female MOAC had better social integration, they had a higher level of social support and thus lower the level of loneliness. A study among the Chinese MOAC revealed that family support mediated the association between acculturation and loneliness [71]. Anna et al.’s study compared foreign-born and native-born people in Sweden and found that lower social integration would generally indicate lower social support, and furtherly caused mental health inequalities between the native and immigrant Swedes [72]. Interaction theory [18] suggested that social support could reinforce social networks and meet the need for social contact, and finally reduce the individual’s loneliness [73]. Social integration could extend the social network of older adults, thus provide more access to social support [74]. This study measured the social integration of female MOAC in four dimensions, including monthly income in the economic dimension, sense of belonging in the psychological dimension, familiarity with the dialect in the cultural dimension, and social participation in the community dimension. An Israeli study revealed that economic status could affect the loneliness of older adults by influencing the perceived level of social support [75]. It was also found that a greater sense of belonging leads to higher perceived social support among individuals [76]. When the female MOAC master the local language and actively participated in community activities, they would have a higher level of social contact with neighbors and friends and receive more social support, which could finally relieve their loneliness [77].

### Implications

Based on the results of this study, the following suggestions are given to improve the loneliness of female MOAC. Firstly, female MOAC could actively integrate into the local community and join more social activities of the community. Secondly, families have the important function of providing emotional support, hence, the children of female MOAC could give more care to their parents and create a good family atmosphere [71]. Thirdly, the community could create a good community environment and provide more social participation opportunities to help female MOAC to integrate into the inflow city better. As the community is the main living place of the elderly migrants and the main platform for their personal interactions, the creation of a friendly and accepting atmosphere is very conducive to the social integration of the elderly migrants [78]. For example, communities should build more public places to provide a convenient environment for MOAC to socialize, exercise, study and relax, and they can also regularly organize group activities and square dances for MOAC [79].

### Limitations

This study had some limitations. Firstly, the data used in this study was from a cross-sectional survey, which could not determine the causal relationship between variables. Secondly, the indicator used to assess social integration were some questions based on previous studies and not measured using a scale, future studies are needed to make the measurement more objective and scientific. Thirdly, the variables used in this study may also be influenced by other confounding factors, thus more research is needed to verify their association.

## Conclusions

As the first research to explore the relationship between social integration, social support, and loneliness in female MOAC, it was found that the female MOAC’s loneliness was at a relatively lower level. Moreover, this study also revealed that social integration and social support were both negatively associated with loneliness, while social support mediated the relationship between social integration and loneliness. To conclude, better social integration and better social support would generally indicate lower loneliness in the female MOAC.

## Data Availability

The datasets used and analyzed in this study are available from the corresponding author upon reasonable request.

## Appendix

**Table 5:** Multi-linear regression analysis between loneliness and other related variables

Characteristics	Model 1		Model 2	
	***P***	**Beta**	***P***	**Beta**
**Age**				
60–65	Ref		Ref	
Over 65	0.067	-0.09	0.038	-0.10
**Education**				
Illiterate	Ref		Ref	
Elementary school	0.145	-0.08	0.111	-0.08
Middle School	0.038	-0.11	0.082	-0.09
High School and above	0.216	-0.06	0.827	-0.01
**Marital status**				
Married	Ref		Ref	
Unmarried	0.486	-0.04	0.836	-0.01
**Chronic diseases**				
No	Ref		Ref	
Yes	0.443	0.04	0.365	0.04
**Self-rated health**				
Excellent	Ref		Ref	
Very good	0.647	0.03	0.684	0.02
Good	0.685	-0.02	0.711	-0.02
Fair and below	0.835	-0.01	0.948	0.00
**Monthly income**				
0–100 RMB			Ref	
101–500 RMB			0.030	0.11
501–1000 RMB			0.299	-0.05
1001–2500 RMB			0.005	-0.14
2501–10000 RMB			0.004	-0.15
**Local Chinese dialect**				
Can’t understand or speak			Ref	
Can understand but can’t speak			0.549	0.06
Can speak a little			0.692	-0.04
Can fluently speak			0.043	-0.10
**Feel like a local**				
Don’t agree			Ref	
Fair			0.888	-0.01
Agree			0.001	-0.19
**Participate in community activities**				
Never			Ref	
Occasionally			0.012	-0.12
Sometimes			0.018	-0.12
Often			0.002	-0.18
**Social support**	< 0.001	-0.30	< 0.001	-0.24

Table 5 showed the results of multi-linear regression analysis between loneliness and other related variables. Model 1only included covariates and social support, and Model 2 added social integration. The results of Model 2 indicated that all four dimensions of social integration and social support were influencing factors of loneliness, controlling for covariates

## Abbreviations

MOAC: The migrant older adults with children
PSUs: Primary sampling units
SSUs: Secondary sampling units
ULS-8: The eight-item version of the University of California Los Angeles Loneliness Scale
SSRS: The Social Support Rating Scale
95% CI: The 95% confidence interval
SEM: Structural equation model
RMSEA: Root mean square error of approximation
IFI: Incremental fit index
GFI: Goodness-of-fit index
AGFI: Adjusted goodness-of-fit index
CFI: Comparative fit index
